# Posterior reversible encephalopathy syndrome in SARS‐CoV‐2 infection: A case report and review of literature

**DOI:** 10.1002/ccr3.7173

**Published:** 2023-04-02

**Authors:** Mahsa Ziaee, Maryam Saeedi, Mohammad Rohani, Masoud Mehrpour, Bahram Haghi Ashtiani, Babak Zamani, Fahimeh Haji Akhoundi, Misagh Salahi Khalaf, Seyyedmohammadsadeq Mirmoeeni, Amirhossein Azari Jafari, Zahra Shateri

**Affiliations:** ^1^ Department of Neurology Firoozgar Hospital, Iran University of Medical Sciences Tehran Iran; ^2^ Department of Neurology Shahroud University of Medical Sciences Shahroud Iran; ^3^ Department of Neurology Hazrat Rasool Hospital, Iran University of Medical Sciences Tehran Iran; ^4^ Department of Neurology Shahid Beheshti Medical University Tehran Iran; ^5^ Student Research Committee, School of Medicine Shahroud University of Medical Sciences Shahroud Iran

**Keywords:** COVID‐19, posterior reversible leukoencephalopathy syndrome, PRES, SARS‐CoV2

## Abstract

Consider PRES in SARS‐CoV‐2 infected patients who develop encephalopathy, seizures or impaired vision; especially if the disease is complicated by respiratory distress and need for mechanical ventilation.

## 1 INTRODUCTION

We are reporting a 44 years old White female who presented with seizures. Her test turned positive for SARS‐CoV‐2 infection. Brain magnetic resonance imaging (MRI) was suggestive of posterior reversible encephalopathy syndrome (PRES). We also reviewed the literature of patients with PRES in the setting of SARS‐CoV‐2 infection to date.

Since its first report in Wuhan, China in December 2019, the SARS‐CoV‐2 went on rapidly to become a pandemic with a relatively high rate of mortality and morbidity.^1^ It has been speculated that the virus can enter host cells via the angiotensin‐converting enzyme‐2 (ACE‐2) receptor, which is expressed in several organs containing endothelial cells.^2^ The respiratory system is typically affected but involvement of other systems is increasingly being described, one of them being the central nervous system (CNS).^3^, ^4^ Herein, we report a patient with severe SARS‐CoV‐2 infection with a history of aplastic anemia who developed encephalopathy, seizures and evidence of posterior reversible encephalopathy syndrome (PRES) on imaging.

## 2 CASE PRESENTATION

A 44 years old White female was brought to the emergency department due to her first episode of generalized tonic–clonic seizure while she was asleep. She had developed fever, myalgia, and dry cough 2 days earlier which she did not seek medical care for. Her past medical history was relevant for aplastic anemia diagnosed 3 years ago and treated with cyclosporine 100 mg/day, danazol, and folic acid. She was waiting for a bone marrow transplantation scheduled on July 2020.

On physical examination, she was febrile (T:39°C) with blood pressure of 130/80 mmHg, heart rate of 100 bpm, respiratory rate of 21/min with oxygen saturation of 89% in room air, and no signs of meningeal irritation. She was lethargic with normal‐sized and reactive pupils, no focal neurologic deficit, and downward plantar reflexes. She was transferred to the intensive care units (ICU) and received levetiracetam to control seizures.

**FIGURE 1:**
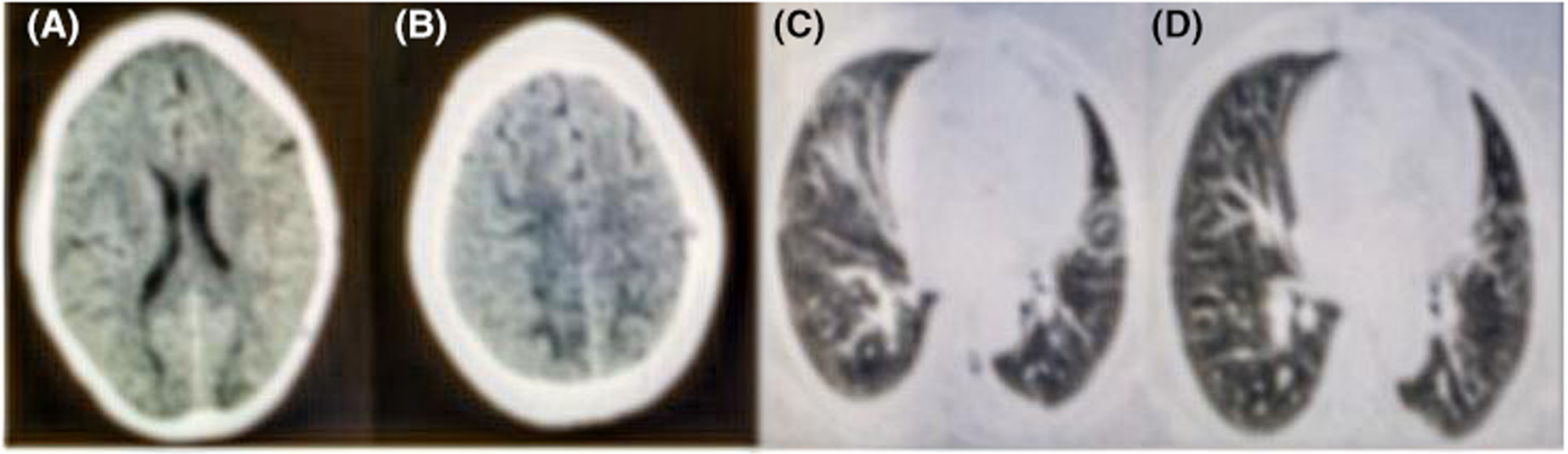
(A, B) Hypointensities in bilateral posterior regions, (C, D) Patchy ground glass opacities.

Initial lab tests revealed white blood cell count (WBC) 900 mm^3^, Hemoglobin 7.3 g/dL, platelet 7000 mm^3^ with normal electrolytes, and biochemistries. Chest computed tomography (CT) scan revealed bilateral multilobar peripheral ground glass opacities in favor of SARS‐CoV‐2 pneumonia (Figure 1).

**FIGURE 2:**
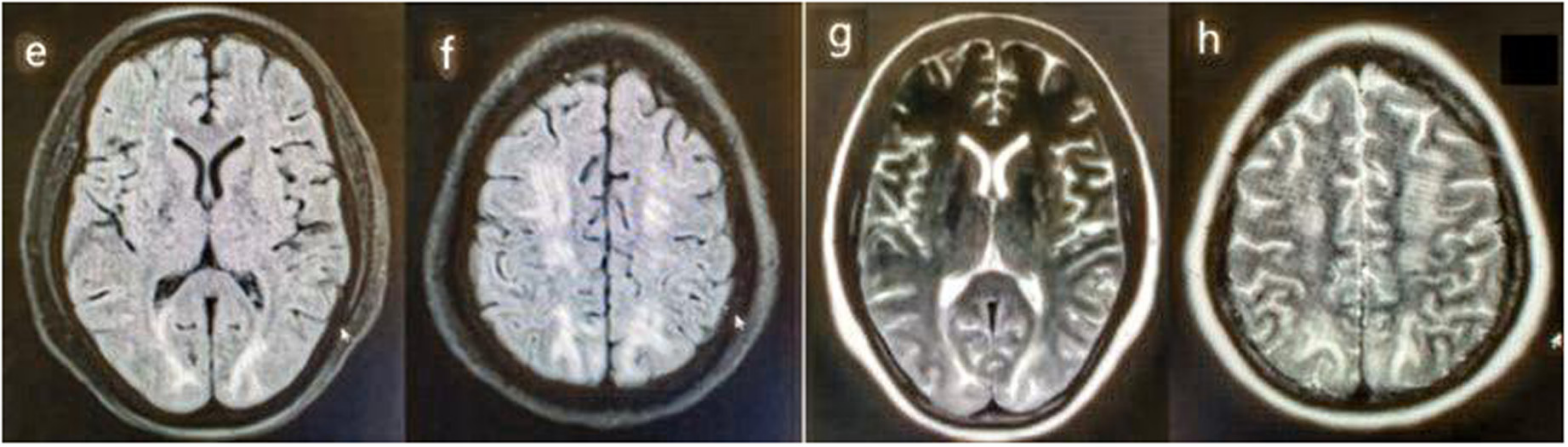
(E, F) Hyperintensities in bilateral occipital in FLAIR, (G, H) Hyperintensities in bilateral occipital and frontal in FLAIR and T2.

Nasopharyngeal real time polymerase chain reaction (RT‐PCR) was positive for SARS‐CoV‐2 infection. Brain CT scan showed hypodensities in posterior regions and brain magnetic resonance imaging (MRI) also displayed subcortical white matter T2/fluid‐attenuated inversion recovery (FLAIR) hypersignalities in bilateral parieto‐occipital regions extending to bifrontal areas, suggestive of PRES (Figure 2). Cyclosporine was discontinued. She was started on steroids (dexamethasone 8 mg three times a day) and meropenem to treat potential concomitant bacterial infection. Treatment with Hydroxychloroquine was not initiated due to a case of PRES associated with its use.

The day after, her respiratory distress worsened and she was placed on invasive mechanical ventilation. Concerning her immunocompromised condition, serologic evaluation for other opportunistic infections including cytomegalovirus (CMV), Toxoplasma, herpes simplex virus, and Epstein–Barr virus was requested and turned negative for acute infection. Cerebrospinal fluid (CSF) evaluation was not done due to thrombocytopenia. Seizures did not recur but after a week she unfortunately passed away due to severe respiratory distress syndrome and cardiac arrest. A brain autopsy was denied by her family.

## 3 DISCUSSION AND CONCLUSIONS

We presented a 44‐year‐old White female with a known history of aplastic anemia who presented with severe SARS‐CoV‐2 infection and CNS involvement in the form of PRES. PRES is a reversible subcortical vasogenic brain edema mostly in the bilateral parieto‐occipital regions, caused by disruption of vascular autoregulation or direct effects of cytokines on the endothelium which results in endothelial dysfunction and breakdown of the blood–brain‐barrier.^5^ It has been described in the setting of eclampsia, renal failure, extreme blood pressure instabilities, cytotoxic or immunosuppressive medications, autoimmune disorders, sepsis, and many other potential causes.^6^ Our patient had a history of aplastic anemia and was receiving cyclosporine. Cyclosporine is a well‐established cytotoxic medication in the etiologic list of PRES^7^, ^8^, ^9^; however, she was clinically stable on cyclosporine for over 3 years. It seems that SARS‐CoV‐2 infection might have pulled the trigger for the development of PRES in this patient. Also it is reported that there is a link between patients suffering from PRES and showing pancytopenia in the lab results.^10^

In a thorough search of the literature and after excluding articles focusing on brain imaging findings and lacking necessary clinical data, we found 18 articles reporting 24 patients with PRES in the setting of SARS‐CoV‐2 infection. Patients' characteristics (including our patient) are presented in Table 1. Subjects were between 25 and 74 years old, with a female predominance (16 women). Seventeen patients (68%) had a preexisting medical condition with hypertension being the most common followed by diabetes mellitus and hyperlipidemia. Two patients were pregnant women at term, and eight had no history of any medical disorders. It has been stated that nearly half of patients with PRES have a history of an autoimmune disorder^5^; however, this was not the case in any of the patients previously reported except for our patient who had a history of aplastic anemia. This finding indicates that the pattern of developing PRES in SARS‐CoV‐2 infected patients is different from other population samples.

**TABLE 1:** Characteristic and demographic data of the similar case reports.

Author	Age/sex	Past medical and drug history	COVID‐19 symptoms		Systemic complications	COVID‐19 treatment	BP mmHg (at onset of PRES)	PRES presentation	Duration between COVID‐19 symptoms and PRES onset (days)	Treatment for PRES	Prognosis
			Systemic	Respiratory							
Agarwal et al.^15^	27/F	Not remarkable	No	No	Acute liver failure	NA	NA	Encephalopathy	NA	NA	Deceased
D'Amore et al.^16^	64/F	Not remarkable	NA	NA	NA	NA	NA	Drowsiness, blurred vision	NA	NA	Improved
Anand et al.^17^	61/F	Not remarkable	Yes	Yes	Sepsis	Remdesivi, anakinra	Max 187/98, labile	Poor mental status, seizure	Day 15 of admission	AED	Improved
	52/F	HIV positive	Yes	Yes	AKI requiring hemodialysis, sepsis	NA	Max 180/97, labile	Seizure	Day 34 of admission	AED	Improved
Cariddi et al.^18^	64/F	HTN, GI reflux, hyperuricemia, hyperlipidemia, OSA, AF	Yes	Yes	Bacterial superinfection	HCQ, darunavir/cobicistat ABs	NA	Drowsiness, blurred vision, lower limbs paresis	35 (day 25 of admission)	None	Improved
Conte et al.^19^	63/F	HTN	Yes	Yes	Moderate AKI, transaminitis	lopinavir/ritonavir ABs, anakinra, heparin	Normal	Seizures	37 (day 30 of admission)	AED	Improved
Djellaoui et al.^20^	69/F	CAD, Hx of endometrial cancer, Hx of breast cancer^b^	Yes	No	None	No treatment	Max 200/116	Seizures, mutism, delirium	7	AED nicardipine	Improved
Franceschi et al.^21^	48/M	Obesity	Yes	Yes	Shock	NA	Max 180/90, labile	Altered mental status	22	NA	Improved
	67/F	HTN, DM, CAD, gout, asthma	NA	No	Mild hyponatremia, mild hyperuricemia	NA	Max 178/83, labile	Altered mental status	NA	NA	Improved
Gómez‐Enjuto et al.^22^	74/M	Multiple myeloma, under treatment with carfilzomib, dexamethasone every 3 weeks for the last 8 months	Yes	Yes	Severe lymphopenia with severe anemia	HCQ, lopinavir/ritonavir, ABs, dexamethasone	Max SBP 150	Status epilepticus, vision loss, paresis	Day 15 of admission	AED verapamil	Improved
Kishfy et al.^23^	58/M	Hyperlipidemia	Yes	Yes	AKI, sepsis, ARDS, Transaminitis	HCQ, ABs, Tocilizumab	Max 189/122, labile	Altered mentation	Day 25 of admission	BP Control	Improved
	67/F	HTN, DM, obesity	Yes	Yes	AKI, sepsis, ARDS, Fungemia, Critical Care Myopathy	HCQ, ABs	Max 193/97, labile	Altered mentation	Day 26 of admission	BP Control	Improved
Llansó et al.^24^	66/F	Not remarkable	‐	Yes	AKI, cardiorespiratory arrest, bacterial superinfection, hyponatremia, hemoptysis	HCQ, lopinavir/ritonavir, ABs, anakinra, tocilizumab	Max SBP 160	Altered mental status	Day 10 of admission	BP Control discontinuing anakinra	Deceased
Mozhdehipanah et al.^25^	39/M	Opium overdose	NA	NA	Transaminitis	HCQ ABs	180/130	Seizures, confusional state, Hemiparesis	3	AED BP Control	Improved but left with hemiparesis
Noro et al.^26^	67/F	Carotid endarterectomy and cardiac arrest^a^	NA	Yes	NA	NA	150/88	Seizure	1	NA	Deceased (due to respiratory involvement)
Parauda et al.^27^	64/M	Not remarkable	No	Yes	AKI, HTN, internal jugular vein thrombosis	HCQ	MAP 128 Peak SBP 187	Encephalopathy, seizure	11	AED BP Control	Improved
	73/M	Not remarkable	No	Yes	AKI, HTN, Multiple infections, MI	HCQ	MAP 135 Peak SBP 212	Encephalopathy and left gaze preference	47(6th week of admission)	AED BP Control	Improved
	65/F	HTN, DM	No	Yes	AKI, HTN, Bacterial pneumonia	HCQ	MAP 138 Peak SBP 190	Stuporous, repetitive blinking	NA	BP Control	Improved
	74/F	Hypothyroidism, DM, hyperlipidemia	Yes	Yes	AKI, HTN, mild transaminitis, persistent respiratory failure	HCQ, tocilizumab	MAP 150 Peak SBP 237	Persistent confusion, intermittent agitation, hemiparesis	NA	BP Control	Improved
Perez et al.^28^	24/F	Pregnant (term)	Yes	Yes	One episode of HTN crises	HCQ, ABs, lopinavir/ritonavir tocilizumab	NA	Hemiparesis, fluctuating levels of consciousness, drowsiness, motor aphasia, agitation	5	AED enoxaparin Methylprednisolone	Partially Improved
Rogg et al.^29^	59/M	Not remarkable	yes	yes	labile HTN	Remdesivir, ABs	Max 173/96	Encephalopathy	Day 12 of admission	NA	Deceased
Sripadma et al.^30^	25/F	Pregnant (term)	Yes	Yes	NA	HCQ, oseltamivir, ABs	Max 190/120	Headache, seizures	2	AED, BP Control benzodiazepines	Improved
Ziaee et al. [this report]	44/F	Aplastic anemia, Cyclosporine	Yes	Yes	Bacterial superinfection	ABs, dexamethasone	130/80	Seizures encephalopathy	2	AED, Dexamethasone, Cyclosporine stopped	Deceased

Abbreviations: AB, antibiotic; AED, anti‐epileptic drug; AF, atrial fibrillation; AKI, acute kidney injury; BP, blood pressure; CAD, coronary artery disease; DM, diabetes mellitus, GI, gastrointestinal; HTN, hypertension; HCQ, hydroxychloroquine, NA, not available; OSA, obstructive sleep apnea; P, positive.

^a^ The patient had gone carotid endarterectomy following an acute carotid stent occlusion 4 days before admission; the surgery was complicated by cardiac arrest that lasted 10 seconds and resolved with no complications.

^b^ The patients had history of endometrial cancer which has been in remission since 2006, and right breast cancer which has been in remission since 2018 and the patient was not receiving any anti‐cancer medication.

About 19 patients (76%) had respiratory involvement of which 18 were in need of mechanical ventilation. The course of hospitalization was further complicated in 18 patients, including acute kidney injury in 10 (40%). Interestingly renal failure has been suggested as the strongest predictor for the development of PRES^5^ and it has been reported in 55% of patients. Six patients developed liver injury, and four cases were complicated by septic shock. Blood pressure recordings above 140/90 mmHg were reported in 18 (72%) patients. Hypertension and respiratory involvement were the strongest predictors of PRES in SARS‐CoV‐2 infected patients.

Fourteen patients (56%) received hydroxychloroquine (HCQ) for COVID treatment. PRES associated with HCQ use has previously been reported.^11^, ^12^ However; it has exclusively been in the setting of systemic lupus erythematous or other immunologic disorders. Whether HCQ plays a role in developing PRES needs further studies. Seven patients (28%) received immune therapies including Anakinra and Tocilizumab. PRES symptoms were detected as early as the first day of SARS‐CoV‐2 presentation until the 6th week throughout admission. The course of PRES was favorable with improvement in 80% of patients.

It is believed that hypoxic ischemic damage to the endothelial cells, excessive cytokines release, immune thrombotic microangiopathy, and direct viral induced endothelial cell injury are accountable for endothelial dysfunction and the resulting cerebral edema and PRES in patients with severe SARS‐CoV‐2 infection. In the setting of an acute inflammatory response, lymphocytes and monocytes produce large amounts of circulating cytokines including tumor necrosis factor α, interleukin 1, and interferon γ, which in turn result in endothelial dysfunction and blood brain barrier breakdown.^5^ Although our patient did not respond to steroid therapy, based on above theories, steroids seem reasonable in the setting of SARS‐CoV‐2‐induced PRES and there is actually one report of good response to them.^13^ It is also worthy to mention that a pattern of complement‐mediated microvascular injury and procoagulant state have been described in the lungs and/or skin of five patients with severe SARS‐CoV‐2 infection.^14^

We presented a patient with a history of aplastic anemia who presented with symptoms of PRES a few days after SARS‐CoV‐2 infection. We also reviewed other reported cases of PRES in the setting of SARS‐CoV‐2 infection. We suggest physicians to consider PRES in SARS‐CoV‐2 infected patients who develop encephalopathy, seizures, or impaired vision; especially if the disease is complicated by respiratory distress and need for mechanical ventilation and labile blood pressure and hypertension. More studies are needed to clarify the mechanism of PRES in SARS‐CoV‐2 infected patients; meanwhile excessive cytokine release and complement mediated endothelial injury are considered accountable.

## AUTHOR CONTRIBUTIONS

**Mahsa Ziaee:** Conceptualization; data curation; writing – original draft; writing – review and editing. **Maryam Saeedi:** Data curation; investigation; resources; writing – original draft; writing – review and editing. **Mohammad Rohani:** Investigation; resources; writing – original draft; writing – review and editing. **Masoud Mehrpour:** Data curation; investigation; writing – original draft; writing – review and editing. **Bahram Haghi Ashtiani:** Investigation; resources; writing – original draft; writing – review and editing. **Babak Zamani:** Investigation; resources; writing – original draft; writing – review and editing. **Misagh Salahi Khalaf:** Investigation; writing – original draft; writing – review and editing. **Zahra Shateri:** Investigation; writing – original draft; writing – review and editing.

## FUNDING INFORMATION

This research received no specific grant from any funding agency in the public, commercial, or not‐for‐profit sectors.

## CONFLICT OF INTEREST STATEMENT

The author(s) declared no potential conflicts of interest with respect to the research, authorship, and/or publication of this article.

## ETHICS APPROVAL AND CONSENT TO PARTICIPATE

Not applicable.

## CONSENT FOR PUBLICATION

Written informed consent was obtained from the patient for publication of this case report and any accompanying images.

## Data Availability

The case report data is not publicly available, but it could be available from the corresponding author with a reasonable request.
